# The Relations of General Medicine to Specific Modes of Medication

**Published:** 1874-09

**Authors:** Alfred Mercer

**Affiliations:** Prof. of Clinical Surgery in the Medical Department of the University of Syracuse, N. Y.


BUFFALO

### Medical and Surgical Journal.

VOL XIV. SEPTEMBER, 1874. No. 2.

### Original Communications.

—:o:—

ART. I.—*The Relations of General—Scientific Medicine—to Special and Specific Modes of Medication.* By ALFRED MERCER, M. D., Prof. of Clinical Surgery in the Medical Department of the University of Syracuse, N. Y.*

*Mr. President and Gentlemen of the Onondaga County Medical Society:*

I believe these resolutions are among the most important ever presented for the consideration of this Society. They dip down deep into the whole subject—into all the relations of medicine and medical science. This discussion involves a vast amount of responsibility—the real status of the medical profession and medical science.

---

*At the semi-annual meeting of the Onondaga Medical Society, held Jan. 28th, 1873, the following resolutions were introduced by Dr. Potter:

WHEREAS, The dictates of reason and common sense commend the fraternization of all regular practitioners of medicine recognized by our State laws, therefore,

*Resolved*, That it is the sense of this Society that the code of ethics be so amended as to be more in harmony with the spirit of our Republican institutions.

*Resolved*, That delegates to, and permanent members of the State Medical Society be requested to urge that body to so amend section 7*th* paragraph *one*, of the code of Medical *ethics*, that members of County Societies may be allowed to meet in *consultation*, all practitioners who are recognized by the laws of the State, whenever called upon by them or their patients.

The Doctor urged his resolution in strong terms, and thought when ministers of the Gospel of different denominations are working together, it was time for physicians to recognize those of other schools, who are gentlemen of culture. Every day the rule was being violated in this city, and he thought physicians should have more liberty in this matter.

Dr. McDonald seconded the resolutions.

Dr. Campbell thought the question was too large to come up so late, and moved to lay it on the table. Carried.

At the quarterly meeting of the Society, held April 8th, 1873, the resolutions were taken from the table, and discussed in the following paper. At the conclusion of the reading of the paper, it was moved by Dr. Plant "That Dr. Mercer be requested to present his interesting paper to the Medical Association of Central New York." The motion was carried.

The paper was read before that Society, June 17th, 1873. At its conclusion a motion was made and carried, requesting a copy for publication.

We feel altogether incompetent to do justice to the occasion, but would beg your indulgence, while we offer our mite of thought for consideration.

But sir, allow me to premise, the medical profession exists not for itself, but for mankind. Sin did not enter the world to create the clerical profession, neither are churches organized for the benefit of the clergy, but to develop, promote and direct the growth of Christian character. Laws are not enacted for the creation and benefit of the legal profession, but for the protection of the citizen. Railroads and telegraphic lines are not constructed for the benefit of the stockholders alone, but for the pleasure and convenience of the public. Mankind are not afflicted with disease solely for the benefit of doctors. The medical profession exists not for itself, but for the good it can do ; and individually, as we are able to alleviate suffering and conduct disease to a successful termination, so will be the honor and usefulness of our profession, so the measure of our success—success in all its phases, in reputation, character, usefulness, and in material reward.

We are the instruments, the workers, the handmaids of medical science, and as such, are comparitively of little importance, but medical science is of great importance. We die, and pass away—medical science lives on—it is its life, progress, and well-being, we are to defend in this discussion—we need take no thought for ourselves, but all thought for the science—and be assured the science will take all needful thought of us.

If these views are correct, we can narrow down the discussion of these resolutions to a single thought, will their adoption tend to advance and promote the diffusion of medical knowledge? For the present, I am of the opinion their adoption would have no such influence; if it can be shown that it would, I shall cheerfully vote for the resolutions.

But Mr. Chairman, I am pleased with the broad and catholic spirit of the resolution, as being in harmony with the spirit of the age, and our republican institutions. On this point, personally, I hope to challenge the living and the dead, as to the freedom of thought, and the equal rights and privileges of all men. In this connection, the Doctor supported his resolutions by referring to the growing liberality of the clergy of different denominations, also to the frequent violation of our code of medical ethics by members of this Society. We fully appreciate this growing liberality of the clergy, and still there is room for more.

The church, past or present, cannot shame medicine in this direction. But the resolutions do not involve liberality and tolerance so much as they involve knowledge, justice and honor. No matter what the clergy may do, these resolutions are to stand or fall on their own merits. They are to be discussed, and disposed of, in the interest of diseased and suffering humanity, and in the special interest of no one else. They are to be carefully considered for the best and for the right, without prejudice, fear or favor. The sick having the first right, and I have so much confidence in the right, that if we look after the interest of the patient, all other interests will be best served.

The occasional violation of the code of medical ethics should be no excuse for their amendment, if they are founded in honor and equity. As well might we clamor for the amendment or repeal of the criminal code, because it is violated every day.

The code of medical ethics is drawn with scrupulous care, and with the deliberate conviction as to its exact justice to all parties concerned. But there are those among us who would throw up the code altogether, and allow every one to keep such professional company as may please him best.

I can hardly agree to this, but times and things may be changing more rapidly than we think for.

The truth and justice embodied in the code of medical ethics, in the light of yesterday, may not be truth and justice in the advancing knowledge of to-day. The code may have outlived its usefulness—it may be dead and effete. That is the question we are to discuss to-day. Good men require little or no law to regulate their conduct. The code is no burden to the honorable and educated physician. Unfortunately, physicians are not all saints, and the code may have some influence in keeping bad men in the line of duty, and I believe it well for us all to be held under some wholesome restraint—to have some standard of etiquette to appeal to for the government of our conduct.

Though I agree with the catholic spirit of the resolutions, I feel that we can better define our own position, and I hope the position of this Society and the profession at large, by offering the following substitute resolution:

*Resolved,* That we recognize all men and women as physicians and surgeons, and will meet them in consultation, if of good moral character, who have made themselves familiar with the following branches of medical science: Anatomy, physiology, general and organic chemistry, symptoms and pathology of medical and surgical diseases, with the principles and practice of midwifery; with the effects of drugs and medicines on the human body in health and disease, and who practice medicine and surgery, under the simple designation of Doctor in Medicine.

And now sir, as an apology for offering a substitute resolution—I am not particular about its adoption as we shall see by-and-by—I desired some starting point, some definition, some platform on which the profession is supposed to stand, some exposition of its present attitude to the whole outside world of medicine.

We propose to discuss these resolutions in a true catholic spirit, with an open heart and a free hand, for we have nothing to conceal or to shun, no malice to ventilate, and no purpose to serve but the good of all; keeping constantly in view the cardinal ideas—the interest of the patient, and the diffusion and advancement of medical knowledge.

We need not occupy your time in pointing out the importance of all the medical knowledge set forth in the substitute resolution to the practicing physician and surgeon. Everywhere, in all the departments of life, knowledge and experience is prefered to ignorance; the practice of medicine forms no exception to the rule. All this knowledge is about equally necessary for one to judge of medical theories and medical practice.

And yet almost everyone feels competent to sit in judgment on these subtle questions. This thought is more or less applicable to the press, in its comments on the recent action of the Massachusetts Medical Society, in expelling some of its members on account of their embracing homeopathy. We are ignorant of the merits of the case and have no comments to make. We mention it that we may say a word or two about the press and ourselves. The press, almost uniformly, comments unfavorably upon us, and the action of our societies. We are always in the wrong, and some one else is always in the right. Now I believe the press aims at the right as well as ourselves, but how comes this almost constant discrepancy? Does the press, and the public, fully understand the situation, or are we really in the wrong? It is to be presumed we understand the situation, and possess the requisite knowledge to judge; but are we really blinded by prejudice as to the right? Does the press form its judgment from actual knowledge of medical subjects or from popular prejudice? If we really are in the wrong I hope we may soon learn the right. We have no time now, specially and specifically, to discuss these questions, but hope incidentally to throw some light on them as we proceed.

For ourselves we are not very particular how or where this requisite knowledge is obtained. It may be possessed without a college diploma, and may possibly be wanting with one. But its possession does not of itself make one a good physician. The best class scholars do not always make the best prescribers, and it has been remarked that "high science may leave one stupid for practice." With proper deportment, and with a knowledge of the branches set forth in the substitute resolution, I should feel disposed to fellowship any man or woman in the practice of medicine.

But before we go further, this Society is not an inquisition, and I shall speak of this Society as a representative society, and synonymous with the profession at large—for membership, it asks no question as to theories, opinions, or belief, but what does the candidate know, and is he of good moral character? This Society, then, is not only a scientific society, but a moral society, moral to the extent of common honor and honesty, and during my connection with it, all expulsions have been for immoral or other unprofessional conduct, altogether outside of medical theory or practice. And in my opinion there is no organization, no society known among men, where there is greater tolerance, greater liberty of thought, and greater freedom of will to do, or not to do, and still hold honorable connection and fellowship, than in our medical societies, and where every man's opinion is respected and deferred to, so long as he grants the same privileges to his fellows. But, when one in arrogance assumes he is right, and all others are wrong, when he claims superior wisdom and judgment, and blazons his ideas to the world as something new and wonderful and beyond the knowledge of other men, to captivate the credulous, and pamper to prejudice and ignorance, he looses cast among us as he ought to do.

It is not so much, then, what the practitioner really believes or does, as how he represents or misrepresents himself and others to the public. We ever have been, and are still charged with being arrogant, bigoted, and intolerant. These charges may, or may not have been true in time past; at present, we are only intolerant of ignorance, and false pretences; and, though we may not all be well learned and all honest, we believe we are as honest as the average of mankind, and that our schools furnish the best, the most thorough and complete medical education that can anywhere be obtained.

The resolutions of Dr. Potter propose to so amend the code of medical ethics that the members of county medical societies may be allowed to meet in consultation all practitioners who are recogniced by the laws of the State. If I understand the laws of the State, there are no laws in force on the subject. Any person may practice medicine and surgery that feels disposed to do so, and the law recognizes him or her as a physician and surgeon. The resolutions then propose that we consult with all forms of quackery and ignorance. In its practical workings will this tend to lower or elevate the standard of professional knowledge, usefulness and respectability?

We cannot spend much time in discussing the question of law, but such is the law, and, professionally, it suits us well enough, but we have some interest in humanity. We are able to take care of ourselves without law. A learned profession needs no special protection; learning always has the advantage of ignorance. For what, and from what, do we need protection? A thorough knowledge of our profession is superior and better protection than any law can be, however cuningly devised. That is the best, and all, the protection we can, or ought, to have. It is the people not the medical profession, that need the protection of law. This absence of law, no doubt, is up to their standard of intelligence, and their ideas of liberty, and it suits us if it suits them. When they are more intelligent, they will ask for protection. The people must move in this matter, not we. If we move, the whole world of quackery and ignorance would be arrayed against it and us, and would cry persecution. Law making, to prescribe the knowledge deemed necessary to practice medicine, must abide its time in the progressive enlightenment of the people.

But laws are in force that attempt to protect the people from the wiles of the sharper, that they shall not be indiscriminately deceived, cheated and killed at the pleasure of cupidity and avarice. Steam boilers are inspected, as well as many articles of food. We have boards of health, and health inspectors. Lawyers are examined before they practice in the courts, and ministers, before they enter the pulpit. But there is no examination, no qualification, deemed necessary in the eyes of the law for one to practice medicine and surgery. This certainly is republican liberty, if not democratic license.

Should not every man that puts out a sign, physician and surgeon, be qualified to stand on the platform we have erected in the substitute resolution, and, as there is no law requiring such qualifications, should we not be a law unto ourselves and to an undiscriminating public, as we are in the by-laws of this Society and in the code of medical ethics?

From our knowledge of the subject, we ought to be the most competent judges of the matter, and, in this, we have no selfish ends in view. For ourselves, simply as medical practitioners—by the means of which we expect to earn a livelihood—it is of little importance whether the original resolutions shall be adopted by this Society, or whether the State shall see fit to set up some medical standard as a prerequisite to the practice of medicine and surgery. All this is of vastly more importance to the public than to us.

Very few possess knowledge sufficient to be judges in medicine, and the outside world of medicine is largely sustained by ignorance and prejudice. Here we may be met with the statement that many very intelligent men—men in the learned professions—select their physicians from this outside world of medicine, but we know of no intelligent man in medicine that does so.

Medical knowledge and medical judgment comes not by intuition, not by reading newspapers and novels, not by studying politics, not by studying Latin and Greek, theology or law, but by studying medical books; and where are the very intelligent men that study medical books? They may be learned and intelligent everywhere else, but unless they are learned in medicine, their medical judgment is worth little more than the judgment of the street boot-black.

Not that the primary branches of medical science are very difficult of comprehension, but to the unlearned they are as a foreign tongue. For instance, we know the Greek from the Roman characters—there our knowledge of Greek ends. We know nothing as to the pronunciation, translation or construction of the language. We could not by any possible means detect the errors of pronunciation or translation of a quack pseudo-professor of Greek. And now, if we should listen to a correct pronunciation and read a correct translation, it would be equally impossible for us to determine which was the true and which was the false without a knowledge of the language. The unlearned in medicine can be as easily imposed upon as the unlearned in Greek. To judge of Greek scholarship, we must have studied the language. To judge of medical theories and practice, we must have mastered the several branches of medical science, and all the better, if we have witnessed their application at the bed-side. To judge critically in medicine one must himself be competent to practice the healing art. Here as elsewhere, without knowledge there can be no intelligent judgment.

But all the cunning and craft of the quack does not belong to the outside world of medicine. It is not every man that has a medical education, and is a member of a county medical society, that is free from the charge. Our fundamental knowledge as medical men does not materially differ. It is the judgment with which this knowledge is used at the bed-side that constitutes the superior physician. He may, or he may not, be quackish in his character, if he assumes to know and do the wonderful, that other men cannot do. If he promises to do what there is no reasonable expectation of performing—perhaps to secure a paying patient—when he knowingly deceives patients and friends, he is running into quackery.

Again, the attending physician is always at the mercy of the consulting physician—so we are sometimes wary who we consult with—it may develop itself in a fulsome display of sympathy and learning, and no matter how judicious the treatment may have been, it is in the power of the consulting physician to create distrust and thus undermine the confidence placed in the attending physician. He was called too late, if this, or that, had been done, or, if he had treated the case from the beginning, things would have been different. All this, in the main, is quackery, for such expressions can seldom be warranted from any known uniform power of medicine to control disease.

The learned then can play the quack as well or better than the unlearned, for quackery is made up alike of deceptions, vain ostentatious boasting, and ignorance.

But the quack in medicine has his victim at the greatest possible advantage. His deeds are done in secret, or at least, are subject to no intelligent criticism. The lawyer may play the quack in his office, but he is sure to be exposed in court by those who are competent to sit in judgment. The medical quack, learned or unlearned, is not likely to come before any such tribunal.

Deception, ignorance, and prejudice, constitute the life-blood of quackery. They are as prominent elements of our civilization as intemperance, and prostitution, evils that this generation will not overcome, and, no doubt, the best way for us to fight quackery is to let it alone, and study to our professional improvement.

Do what we may, be assured, that, for ages to come, the harvest will be continually ripening for the golden sickle of the audacious charlatan. Greater the audacity, the greater the harvest. Gold is light in the balance to the dying man.

These resolutions propose to grant us the privilege of consulting with at least the following list of doctors: The Clairvoyant, Spiritualist, Uriscopic, Indian, "Heathen Chinee," Botanic, Analytical, Eclectic, Thomsonian, Hydropathic, Homeopathic, Traveling Specialist and non descript mountebanks in general. Liberty enough. I hope the prospect pleases, gentlemen, in the opening up of these Elysian fields. But perhaps the doctor did not intend to include all this array of names in his resolutions. If he did not, where shall the line be drawn, who shall be elected, and who rejected?

Our substitute resolution defines who, and what, we will recognize, and we believe it is broad and liberal enough to include all men worthy of the title of doctor, or worthy of the confidence of the public. It requires ample knowledge of the elementary brances of medical science, leaving each for himself to adopt any theory, or, no theory, and prescribe any medicine, in any dose, that to him may seem best. He is simply to be a doctor, and blow no trumpet of his own superior knowledge, skill, or fame.

We do not propose to examine the status of more than two or three of this long list of doctors, with something added to their names, as each being superior to all other modes of medication.

And first of the Thomsonian, who is now nearly obsolete, but he was a clamerous and an agressive fellow in his day—as we remember him—and captured his patients and disease by storm or "blowing steam," it made little difference which, and is to be remembered mostly for his useful efforts in expunging the last remaining law from the statute book, regulating the practice of medicine.

Notwithstanding the imposing cognomen, Eclectic Physician, it is for the most part simply a clap-trap, catch-penny institution, as being something different from other medical men. His sign is a libel on his declaration of principles,* and should read Discarding Physician in place of Eclectic Physician. If he is what he claims to be, he is one of us, and would be with us. We elect the good and useful, in our judgment, from all sources, and compel nothing. But he wishes the world to understand that he is something different and better than other medical men.

Men put up signs and advertise to exalt, not to demean themselves and their wares before their patrons and the public. But as a class I believe they are improving in medical culture and intelligence.

The botanic sign caters to a prejudice, that it aims to create and

*Transaction of the Eclectic Med. Soc. of the State of N. Y., 1872, page 3.

perpetuate. Its hobby-horse is apt to be calomel, quinine and arsenic—quinine being a vegetable—and that vegetables are all innocious and minerals all poisons. This may be a strong picture, but I believe I have heard it in substance. If it were true, but it is not, we have no law or rule requiring or compelling us to use any mineral remedy whatever. We may use soda, calomel, iron or sulphur, or we may let them all alone and use nothing but vegetables, and still hold honorable fellowship in this society.

The homeopathic sign has its special attractions, especially for children. The medicine is so easy to take, and this is the more common argument used in its favor. We are all supposed to be familiar with Hahnemann's theory of medicine, and there are fewer objections, perhaps, to our consulting with one of his *true* followers, *if such can be found,* than with any other class of men. We could consult with him on moral grounds if not on scientific. He is not a quackish deceiver; he is what he pretends to be. He is simply deceived, or we are. He attributes the recovery of his patients to his thousandth dilutions, we to rest, regimen and nature.

If we consult with him it will stand something like this: First, we should expect a fee, and, it seems to me, this is the hub of the resolutions. They look more to loaves and fishes than to any other interest. Second, we might agree or disagree on diagnosis, prognosis, and treatment. Most likely, we should disagree on treatment, and we might not, for the patient might not require any special or specific medication Our opinion, if favorable to recovery might afford the patient some consolation. I believe this to be the most honorable consultation we can hold outside of the regular profession. But, for all this, humanity first, profession second, to do good in emergencies when called on, but not in the way of formal consultation. But in regard to those who wear the "livery" of Hahnemann and are not his disciples; they are simply hypocrites, using ordinary forces in nature and attributing the effects that follow to their magic wand. Is it desirable that we consult with this form of deception?

And now, in due candor and honesty of purpose, from medicines found, from time to time at the bed side of their patients, from the statements made by their patients themselves, and the druggists of whom they buy their remedies, and from conversation with their own members, we have reason to believe that a very large majority of those styling themselves Homeopathic doctors are in the constant habit of prescribing medicines in ordinary doses, and on ordinary principles of practice, just as we do.

Some of these more honest than others have taken a step in the right direction, and have stricken Homeopathy from their signs, but they still adhear to the organization, though Homeopathic only in name. Why practice this deception on the public? Why keep up this factitious opposition, but to gain sympathy and thus gain patients, and keep up the cry persecution, bigot, intolerant, martyr? The chasm between us and this class of Homeopaths, and we believe it embraces nearly all of them, is but a step, and that step is deception, and I believe is ere long to be more or less perfectly bridged over with truth and honesty. We differ as much, or more, among ourselves than they differ from us as a whole on matters pertaining to theory and practice, still we live in peace, tolerant of each other.

Medical science bears on its front the olive branch of peace and good will to man, and now will some one of our many critics, the press if you please, be kind enough to point out the way of honorable reconciliation between us and any, or all, of of these exclusive systems of medicine? What shall we do, what *pathy* shall we adopt to make friends and secure peace? We will wait the reply of the outside world of medicine, their champions and defenders, and in the mean time allow us to suggest that the chasm may be bridged any day by obtaining a good medical education, keeping special dogmas and pathies in the background, be doctors in medicine simply, standing on professional knowledge and merit for patronage and preferment, granting the right of private judgment to all, as to medicine and dose to be used to relieve disease, and we meet at once as brothers at the bed-side. What is there of bigotry and intolerance in this? If we critically examine the status of medicine, outside the regular profession, there is no fold where an honorable and educated man can find a resting place. They all voluntarily place themselves under bonds before the public—Eclectics excepted—to do or not to do certain things. If they abide by the contract thus made, they so imperfectly represent the curative powers of the healing art that we could scarcely recognize them as brothers. If they disregard the contract, their practice is a fraud and a cheat, and as such, we, certainly, could not recognize them.

With us it is different, we are pledged to nothing, only intelligence and honor. We have set no stakes either in theory or practice beyond which we may not go—we have made no contract—placed ourselves in no straight jacket before the public by adding a prefix to our signs, our thoughts and deeds are as free as the air we breathe.

Again, all honorable and educated medical men may become members of this Society in good standing, under our present laws and ethics without any amendments whatever. Knowledge and good moral character are the requisites to admission, no questions are raised as to theories, medicines to be used, or doses to be administered. We are all left perfectly free on all these points. Who then have we discarded? No one but the ignorant and immoral man, and these men would have the world believe they are martyrs.

As a member of this Society, I am at liberty to believe the Hahnemann theory and administer any medicine in any attenuation I please, and this is all there is of Homeopathy, *and for this alone* there is no law of this Society under which I can be brought to an account; *and for this alone* there is no thought in the code of medical ethics that can be distorted into an excuse for a repremand. The issue then is something outside of medical theory and medical practice. Patients seldom ask what medicines we propose to use, and seldom, if ever, what doses we propose to administer. We give large or small doses, next to nothing, as we think best, and in accordance with the theory of Hahnemann, Brown or Bronssa, or without any theory whatever, only the theory of empirical experience if you please.

If then we are as free to believe and to do as all this, what need we of any resolutions, our borders are large enough already to embrace all honorable and educated medical men.

But all these outsiders set themselves up to be our betters—if they were our commons they would come into our fold—and claim specially and specifically to be our superiors, with new and better modes of practice to secure patronage, for no one intentionally employs an incompetent physician. We are berated as inferiors on all proper occasions, as behind the age, worse than old fogies, our treatment of disease is worse than useless, for it inflicts untold evils on mankind. If we are half as bad as represented, they ought to flee our company and thank heaven for deliverance; they ought to rejoice, as honest men, that they can escape from such a Society, with pure hearts and clean hands, in place of crying persecution, martyrdom, to excite sympathy if they are excommunicated. What sane Society, however villanous or holy, would foster an assassin in its bosom, that was daily thrusting a dagger at its heart.

But, perhaps, after all, it may be necessary to have all these sorts of doctors to please the whims and prejudices of the people—prejudicices and whims that have been mostly excited by denouncing us, whether it is necessary or not, we do not propose to quarrel with them, the most we aim at in this discussion is to point out some of the relations they sustain to legitimate medicine.

We can hardly expect mankind to agree in medicine, while they differ so much in theology, law, government, engineering, temperance, and in nearly all the affairs of life.

But, there can be only one true science of medicine, and that, in the details of practice, must be infinitely variable, to meet the exigencies of constitutional peculiarities, age, sex, climate, occupation, endemic epidemic, and other influences. There must also be a one best way to treat disease, not that inferior treatment does not often succeed, and there are many very serious cases of disease that terminate in health without any treatment whatever. But in the whole medical world as it now stands, in what direction ought we to look, expecting to find this one best treatment. Among the learned, or among the ignorant, among those who have tied their hands, and set limits to medicine and medication, to work inside a theory or dogma, or among those who encourage the most learning and exercise the greatest possible freedom of thought and action. Let us carefully review the situation, and, if we are not in this one best line of direction, let us hasten to find it, summoning all human wisdom and experience to our aid. Have we been on the wrong track, have we really been napping in our activity and zeal in prosecuting medical inquiry, and now wake up to find ourselves second in medicine? If we are in this unfortunate condition, let us scramble to the front before we offer our council to those in advance. If we are at the front, is it well to look back while there is so much unoccupied territory before us? We ever welcome honor and learning to our standard; here is congenial soil for both to flourish. All the world of medicine can come to us in honor, but to what ism or pathy can we go and maintain our honor and self respect?

We have said little, or nothing, as to the merits or demerits of these outside systems of medicine at the bed-side. We have directed attention more to the relations they sustain to medical science, and the well-being and protection of the public against fraud and deception, for the reason, that practical medicine is one of the most abstruse subjects that can well engage the attention of the human understanding. It undertakes to handle, manipulate, guide, control, or direct, by material agency, almost—shall I not say an immaterial force—certainly a force that science does not comprehend, and over which these material agents have no absolute uniform and constant control.

In disease, we are cognizant of certain changes taking place in the functions or structure of one or more of the various organs composing the human body. To devine the cause, the same apparant cause often producing very different diseases, owing to constitutional differences and predispositions, to interpret the signs manifested by these diseased organs, to direct remedies to so modify vital force as to restore them to health, is the special business of the physician. But medicines do not fit names and diseases by any uniform and absolute rule as a tenon fits a mortice, or a keystone fits an arch—the same exposure is not always sure to induce the same disease. And now this vital force is always varying, and always an unknown quality, that no symbol can represent, therefore we have no fixed positive data, certainly in one direction to reason from in practical medicine. Thus comes the abstruseness of the subject.

With these elements of the unknown and apparently the unknowable, reason is the sport of uncertainty, and the best observers are baffled in judgment to give due credit to each—vital force and medicine—in the successful management of disease. But this vital force is always present, often very feeble, with its best endeavors to restore the diseased organism to health.

And in this connection, if we may venture an opinion, it would be that in general we overestimate the importance of medicine in the cure of disease. We know that very many diseases are self-limited in this nature, and tend to terminate in health without medication.

"To know the natural cure of disease is more than half of therapeutics." The reputation of the inert materia medica of one or two hundred years ago; the reported cures by charms, amulets, metalic tracters, royal touch and the fairy globules of to-day, all point in this direction. And when we contrast all this with the heroic treatment of fifty years ago—the enormous blood-lettings, and other active perturbative treatment, and inquire as to the result of these two extremes of practice, I believe nature may fairly dispute for honors among all the doctors.

In all this I hope not to undervalue the powers and usefulness of the healing art; we often witness its significance, and in heroic practice to, but the secret is to know when to do, when to trust nature, and when to resort to art. This is the one great lesson we all want to learn.

Now all these outside systems imperfectly represent medical science, and often misrepresent it by charging it with many faults of which it is not guilty, exciting a prejudice in their own favor, charging medicine as doing more mischief in breaking down general health than disease itself, of which the whole human family finally perish. Disease is innocent, medicine is all powerful for evil to those who recover. As well might we claim fire to be innocent, and charge all the damage to the water that extinguishes it. They are all partial and circumscribed in their usefulness, but they serve to enlarge the field of medical observation, and we aim to profit by their experiments and experience, and appropriate to our own use what is found useful. We are not particular whence our knowledge.

But, while we may have learned something from these outside systems of medicine, the great and important discoveries in medicine and surgery, the real substantial progress of the science, has been acheived by the regular profession. I may not be well informed on the subject, but I am unacquainted with any really valuable addition made to our stock of knowledge from this quarter, nothing worthy of notice beyond what we believe to be the hypothetical theory of Hahnemann, and whatever of truth may be claimed for it, and we think that very little, two things are certainly true in regard to it. Diseases are successfully treated outside of this theory, and it is not applicable to all forms of bodily ailments.

It is seldom, patients and friends have the right sort of confidence in their medical advisers; they too often think they must be dosed to recover. The sick should be under intelligent medical observation; but often medicine is of little or no service to them.

I have occasionally received a call something like this: Doctor, my daughter, Jennie, does not feel well; we do not know what ails her; I wish you would go and see her, and tell us what the matter is, and if you think she will get along without medicine, you need not give her any. I will pay you for your visit all the same.

This is music to the physician's ear; it has the ring of true metal, and illustrates the relations that should exist between patient and physician. If this was the common relation of physician and patient, the sick would take much less medicine than they do. But we all know how the mind acts and reacts on the body, and that must be taken care of to facilitate recovery, and can be done as well with sugar as with bread pills.

And now, after this imperfect and, perhaps, somewhat irrelevant review of the situation, where is the line of duty? Many of the men practicing medicine outside of the regular profession are supremely ignorant, others, perhaps with more learning, are equally cheats and charlatans. We cannot descend to this sort of fellowship. But there are still others, respectable in society, and fairly respectable in medical knowledge, and what is our duty to them, to medical science, and to the public? From all that we have said and from all that we have thought on the subject, I believe it is our duty to remain just as we are, and for one simple reason among many others that might be adduced. They do not entertain a medical thought that I may not entertain, they never have, and I believe they never will make, a prescription that I might not make and still hold an honorable connection with this Society, with all its privileges and immunities, and they can do likewise if they will. The door is wide open for all honorable and educated men to come in.*

* Since this article was written, an act has passed the Legislature, regulating the practice of medicine, which I think very defective—better than nothing perhaps, for it may lead to something better, since public attention is directed to the subject. If the act had prescribed a form for the examinations for all Societies to follow, and the results of all such examinations had been submitted to some competent board of State censors for approval, the law then might have had some virtue in it, but as it is, I fear it will afford little or no protection to the people from ignorance in the profession, and on the whole have a damaging influence on the general standing of the profession, by giving legal authority to ignorant and unworthy men. At any rate it is liable to this sort of abuse. ALFRED MERCER.

